# Extracorporeal Shock Wave Therapy for the Treatment of Osteoarthritis: A Systematic Review and Meta-Analysis

**DOI:** 10.1155/2020/1907821

**Published:** 2020-03-18

**Authors:** Lu Chen, Ling Ye, Hui Liu, Pingliang Yang, Bangxiang Yang

**Affiliations:** ^1^Department of Pain Management, West China Hospital, Sichuan University, Chengdu 610041, China; ^2^Department of Anesthesiology, The First Affiliated Hospital of Chengdu Medical College, Xindu, Sichuan 610500, China

## Abstract

**Background:**

Osteoarthritis is the most common musculoskeletal disease. Extracorporeal shockwave therapy had shown an effect on osteoarthritis in both some animal experiments and clinical studies, but there was no systematic review to confirm the value of shockwave therapy in the treatment of all types of osteoarthritis and compare it with other traditional therapies (especially traditional Chinese medicine).

**Method:**

PubMed, Medline, the Cochrane Central Register of Controlled Trials, Web of Science, Chinese National Knowledge Infrastructure, WANFANG database, and VIP database were searched up to December 10, 2019, to identify randomized controlled trials comparing shockwave therapy and other treatments for osteoarthritis. Visual analogue scale and the Western Ontario and McMaster Universities Osteoarthritis Index were extracted and analyzed by RevMan and STATA software as outcomes of pain reduction and functional improvement. Adverse reactions were recorded to evaluate the safety of shockwave therapy.

**Results:**

Shockwave therapy had significant improvement in both pain reduction and functional improvement compared with placebo, corticosteroid, hyaluronic acid, medication, and ultrasound (*P* < 0.05). In functional improvement, shockwave therapy showed statistical improvement compared with kinesiotherapy and moxibustion (*P* < 0.05) but not with acupotomy surgery (*P* = 0.24). A significant difference between shockwave therapy and platelet-rich plasma was observed in pain reduction (*P* < 0.05) but not in functional improvement (*P* = 0.89). Meanwhile, a statistical difference was found between shockwave therapy and fumigation in functional improvement (*P* < 0.05) but not in pain reduction (*P* = 0.26). Additionally, there was no statistically significant difference between shockwave therapy and manipulation in both pain reduction (*P* = 0.21) and functional improvement (*P* = 0.45). No serious adverse reaction occurred in all of studies.

**Conclusions:**

Extracorporeal shockwave therapy could be recommended in the treatment of osteoarthritis as a noninvasive therapy with safety and effectiveness, but the grade of recommendations needs to be discussed in a further study.

## 1. Background

Osteoarthritis (OA) is the most common musculoskeletal disease, ranking as the 11th highest contributor to global disability and 38th highest in the disability-adjusted life years (DALYs) in the Global Burden of Disease 2010 study [1, 2]. About 18% of women and 10% of men over 60 years of age suffered from OA and had higher mortality rates than their peers [3, 4]. In recent studies, the pathological processes of OA involve several local and systemic factors such as cytokines, chemokines, inflammatory mediators, matrix degradation, cell-derived, and/or matrix-derived products, which may cause damages to the synovium, cartilage, subchondral bone, periarticular muscles, ligaments, and other joint structures and finally lead to pain, stiffness, and disability [5, 6]. At present, the medical management of OA includes surgical therapies and nonsurgical therapies such as intra-articular injection, medication, and physical therapy. However, it was still difficult to reverse the destruction of joint structures [5]. Therefore, it is of great clinic significance to find an ideal method to relieve pain, improve function, and delay the disease progression.

As a new technique, extracorporeal shockwave therapy (ESWT) uses a single-impulse transient acoustic wave induced by pneumatic, electrohydraulic, electromagnetic, or piezoelectric generators which focuse on the area needed to be treated [7]. ESWT has shown an effect on articular cartilage and subchondral bone development, neovascularization, tissue regeneration, and inflammatory response in some animal experiments [8–10]. ESWT also succeeds in the treatment of several musculoskeletal diseases, including tennis elbow syndrome, plantar fasciitis, tendon disease, and fracture nonunions, in some clinical studies [11–14]. More and more attention has been paid to the application of ESWT on OA because of its noninvasive nature, low rate of complications, and low cost compared with other surgical or conservative treatments in recent studies [15, 16]. Despite some systematic reviews focusing on the effect of ESWT on knee OA [17–19], there was no systematic review to confirm the value of EWST in the treatment of all types of OA (including knee OA and carpometacarpal joint OA) and compare ESWT with other traditional therapies (especially traditional Chinese medicine). Thus, this meta-analysis was performed, and the latest randomized controlled trials were included, which would contribute to the treatment of OA.

## 2. Method

### 2.1. Search Strategy

The protocol was registered in the PROSPERO database (CRD42019120534), and all searched results were evaluated according to the PRISMA statement. PubMed, MEDLINE, the Cochrane Central Register of Controlled Trials, Web of Science (WOS), Chinese National Knowledge Infrastructure (CNKI), WANFANG database, and VIP database were searched up to December 10, 2019, to identify the potential studies exploring the effect of ESWT for the treatment of OA. The searching strategy used was as follows: (((extracorporeal shock wave therapy [Title/Abstract]) OR ESWT[Title/Abstract])) AND ((osteoarthritis[Title/Abstract]) OR OA[Title/Abstract]) Filters: Publication date to 2019/12/10. The publication language was limited to English and Chinese.

### 2.2. Study Selection

The inclusion criteria were the following: (1) randomized controlled trials (RCT) comparing the effect of ESWT and other treatments (including placebo) for all types of OA; (2) full text available and the outcome of experiments including mean (M), standard deviation (SD), and number (*N*); (3) patients aged 45 years or more and diagnosed with OA according to any clinical criteria; and (4) ESWT that had never been performed to the enrolled patients before.

The exclusion criteria were the following: (1) meta-analyses, reviews, letters, editorials, expert opinions, case reports, and nonrandomized control trials; (2) animal experiments; (3) patients with coagulopathy, pregnancy, cancer, history of fractures, cardiac pacemaker use, and neurologic conditions; and (4) including only the latest information if data were duplicated or overlapped.

### 2.3. Screening and Data Collection

Two researchers independently assessed the eligibility of the studies, and the disagreements were resolved by a third verdict. Titles and abstracts were screened to identify the related studies, and then full texts were assessed carefully. Moreover, the references cited in the selected articles were explored to identify the potentially relevant studies. The scores of visual analogue scale (VAS) were extracted as primary outcome. Secondary outcomes included the scores of the Western Ontario and McMaster Universities Osteoarthritis Index (WOMAC), which represented the functional change. If the scores were recorded in different follow-up times, we selected the time point at 3 months or available data to be nearest to 3 months to predict the efficacy.

### 2.4. Quality Assessment

The quality of included studies was assessed by the Cochrane Collaboration's tool for assessing the risk of bias which was recommended for systematic reviews of interventions in Cochrane Handbook version 5.1.0 [20]. We evaluated 7 domains of bias including selection bias, performance bias, detection bias, attribution bias, reporting bias, and other sources of bias. The judgements were expressed as “high risk,” “low risk,” or “unclear risk,” and the quality assessment figure was generated by RevMan version 5.3.

### 2.5. Statistical Analyses

Meta-analysis Review Manager software (RevMan version 5.3; The Cochrane Collaboration 2014) and STATA (version 12.0; Stata Corporation) were used for data analysis. The analysis was performed in two respects including pain reduction and functional improvement. The heterogeneity was evaluated by Higgins I2 statistic, *I*^2^ > 50% was defined as significant heterogeneity among studies, and the random effects model was applied for the pooled effect estimates. Otherwise, the fixed effects model was used. At the same time, subgroup analysis was used for exploring sources of heterogeneity and reassessing the results. Sensitivity analyses were performed by removing an individual study from the meta-analysis each time. If more than 10 studies were included in each meta-analysis, the possibility of publication bias would be evaluated by Egger's test and *P* < 0.05 was considered statistically significant; then the fill method and nonparametric trim were applied to correct the effect size. The results were expressed as the standard mean difference (SMD) and 95% confidence interval (95% CI) for continuous outcome data.

## 3. Result

### 3.1. Search Results

**Figure 1:**
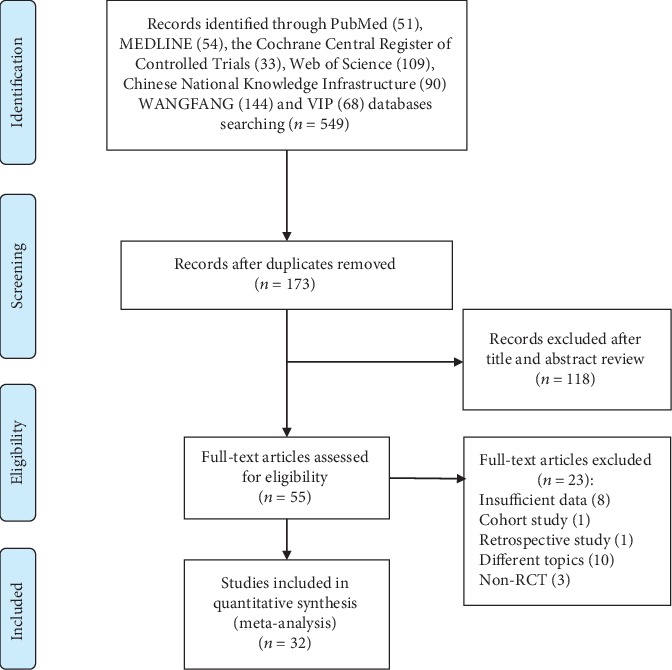
Flow diagram of study selection in this systematic review.

As shown in Figure 1, the initial search yielded 549 articles and 173 records were screened after removing duplicates. The title and abstract of potentially relevant studies were read carefully, and 118 records were excluded. Then 55 full-text articles were assessed, and 23 articles were excluded because they did not meet the inclusion criteria. Finally, 32 RCTs were included in this meta-analysis [21–52]. Characteristics of these studies are shown in Table 1. All of the articles were published between 2013 and 2019 in English or Chinese. The sample size ranged from 18 to 160. All experimental groups received ESWT, while control groups received different treatments including placebo [22, 23, 25, 26, 28, 30, 34, 48, 49, 51, 52], medication [31, 32, 43, 44, 50], intra-articular injections [21, 26, 27, 29, 35, 36, 39, 40], traditional Chinese medicine [38, 41, 42, 45, 46], ultrasound [22, 24, 47], surgery [33], and kinesiotherapy (KIN) [37].

### 3.2. ESWT vs. Placebo

**Figure 2:**
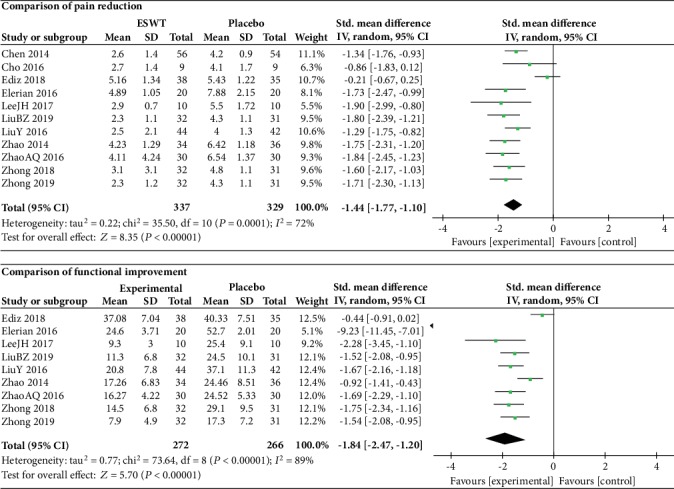
Forest plot comparing the ESWT group with the placebo group.

A statistically significant difference between ESWT group and placebo group was found in pain reduction (SMD = ‐1.44, 95% CI: -1.77 to -1.10, *P* < 0.00001) and functional improvement (SMD = ‐1.84, 95% CI: -2.47 to -1.20, *P* < 0.00001). As shown in Figure 2, high heterogeneity was observed in the analysis of pain reduction (*I*^2^ = 72%). After removing a study [25] from the meta-analysis, the heterogeneity decreased to 0%. The same phenomenon occurred in the analysis of functional improvement; the heterogeneity decreased from 89% to 30% after removing two studies [25, 26] from the meta-analysis, which suggested these two studies might be the sources of heterogeneity. The pooled effect did not change after removing these studies (*P* < 0.00001), which indicated the result was robust.

### 3.3. ESWT vs. Intra-Articular Injections

**Figure 3:**
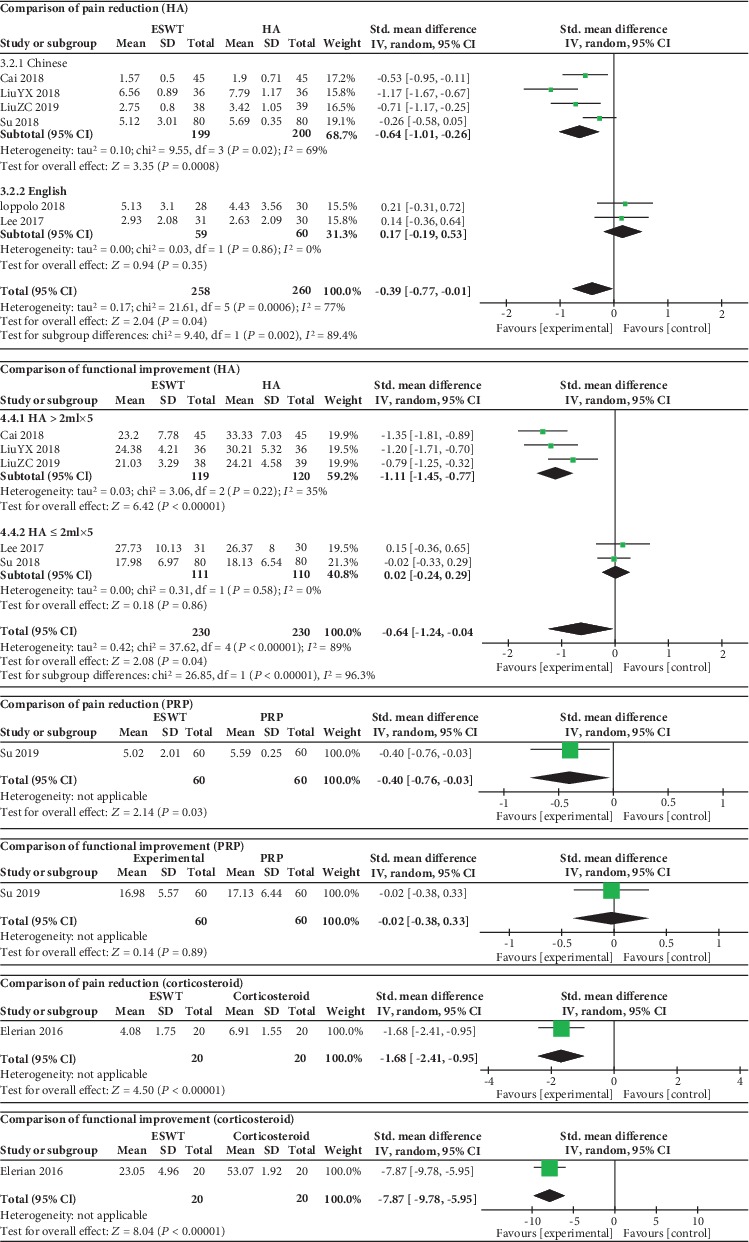
Forest plot comparing the ESWT group with the intra-articular injection group.

As shown in Figure 3, there was a statistical difference between the ESWT group and hyaluronic acid intra-articular injection (HA) group in pain reduction (SMD = ‐0.39, 95% CI: -0.77 to -0.01, *P* = 0.04) and functional improvements (SMD = ‐0.64, 95% CI: -1.24 to -0.04, *P* = 0.04). The heterogeneity decreased after subgroup analysis, which suggested that the language and dose of HA might be potential sources of heterogeneity.

A statistically significant difference between the ESWT group and platelet-rich plasma (PRP) intra-articular injection group was observed in pain reduction (SMD = ‐0.40, 95% CI: -0.76 to -0.03, *P* = 0.03). However, there was no statistically significant difference in functional improvement (SMD = ‐0.02, 95% CI: -0.38 to 0.33, *P* = 0.89).

There was a statistically significant difference between the ESWT group and corticosteroid intra-articular injection group in pain reduction (SMD = ‐1.68, 95% CI: -2.41 to -0.95, *P* < 0.00001) and functional improvements (SMD = ‐7.87, 95% CI: -9.78 to -5.95, *P* < 0.00001).

### 3.4. ESWT vs. Medication

**Figure 4:**
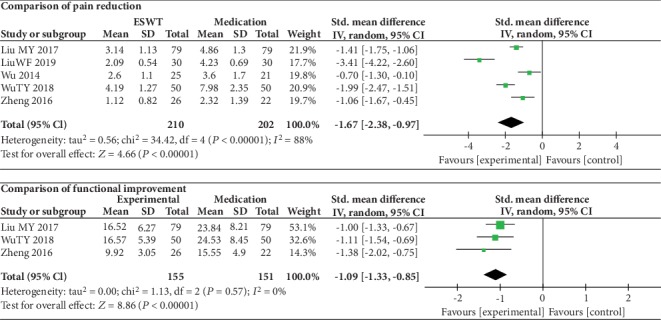
Forest plot comparing the ESWT group with the medication group.

There was a statistically significant difference between the ESWT group and medication group in the pain reduction (SMD = ‐1.67, 95% CI: -2.38 to -0.97, *P* < 0.00001) and functional improvement (SMD = ‐1.09, 95% CI: -1.33 to -0.85, *P* < 0.00001). High heterogeneity was found in pain reduction (*I*^2^ = 88%). In functional improvement, no heterogeneity was observed (*I*^2^ = 0%) (Figure 4).

### 3.5. ESWT vs. Ultrasound

**Figure 5:**
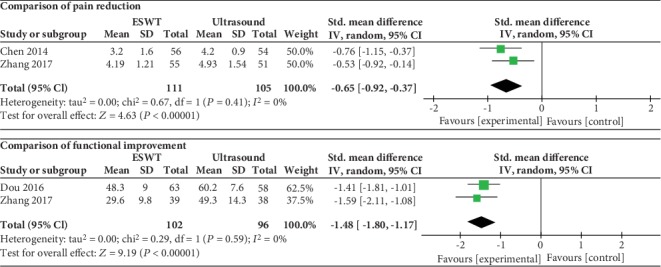
Forest plot comparing the ESWT group with the ultrasound group.

As shown in Figure 5, a statistically significant difference was observed between the ESWT group and ultrasound group in pain reduction (SMD = ‐0.65, 95% CI: -0.92 to -0.37, *P* < 0.00001) and functional improvement (SMD = ‐1.48, 95% CI: -1.80 to -1.17, *P* < 0.00001). No heterogeneity was observed in this meta-analysis (*I*^2^ = 0%).

### 3.6. ESWT vs. Surgery

**Figure 6:**
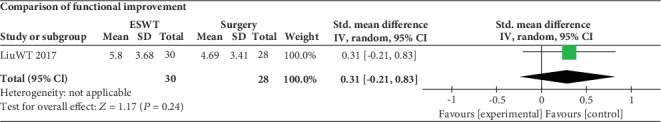
Forest plot comparing the ESWT group with the surgery group.

There was no statistically significant difference between the ESWT group and acupotomy surgery group in functional improvement (SMD = 0.31, 95% CI: -0.21 to 0.83, *P* = 0.24). (Figure 6)

### 3.7. ESWT vs. KIN

**Figure 7:**
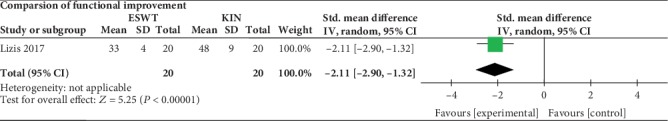
Forest plot comparing the ESWT group with the KIN group.

In Figure 7, a statistically significant difference was observed between the ESWT group and kinesiotherapy (KIN) group in functional improvement (SMD = ‐2.11, 95% CI: -2.90 to -1.32, *P* < 0.00001).

### 3.8. ESWT vs. Traditional Chinese Medicine

**Figure 8:**
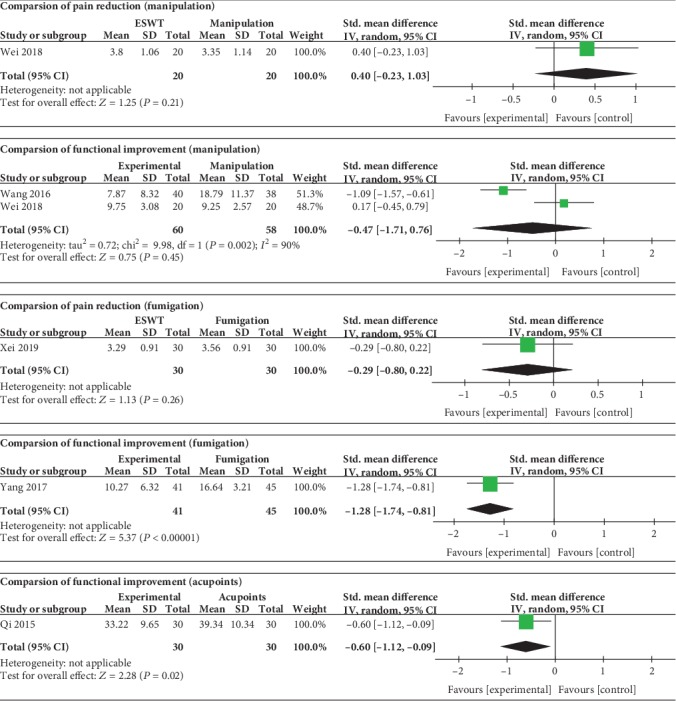
Forest plot comparing the ESWT group with the traditional Chinese medicine group.

As shown in Figure 8, there was no statistically significant difference between the ESWT group and manipulation group in pain reduction (SMD = 0.40, 95% CI: -0.23 to 1.03, *P* = 0.21) and functional improvement (SMD = ‐0.47, 95% CI: -1.71 to 0.76, *P* = 0.45). A statistically significant difference was found in comparison between the ESWT group and fumigation group in functional improvement (SMD = ‐1.28, 95% CI: -1.74 to -0.81, *P* < 0.00001) but not in pain reduction (SMD = ‐0.29, 95% CI: -0.80 to 0.22, *P* = 0.26). There was a statistically significant difference between the ESWT group and acupoint moxibustion group in functional improvement (SMD = ‐0.60, 95% CI: -1.12 to -0.09, *P* = 0.02).

### 3.9. Adverse Event

Only temporary pain, minor bruising, or transient soft tissue swelling was observed in nine studies [25, 30, 34, 42, 44, 47, 50–52]. No adverse events were observed during the treatment in other six studies [27, 29, 36–38, 46], and the remaining studies did not mention it.

### 3.10. Sensitivity Analysis

**Figure 9:**
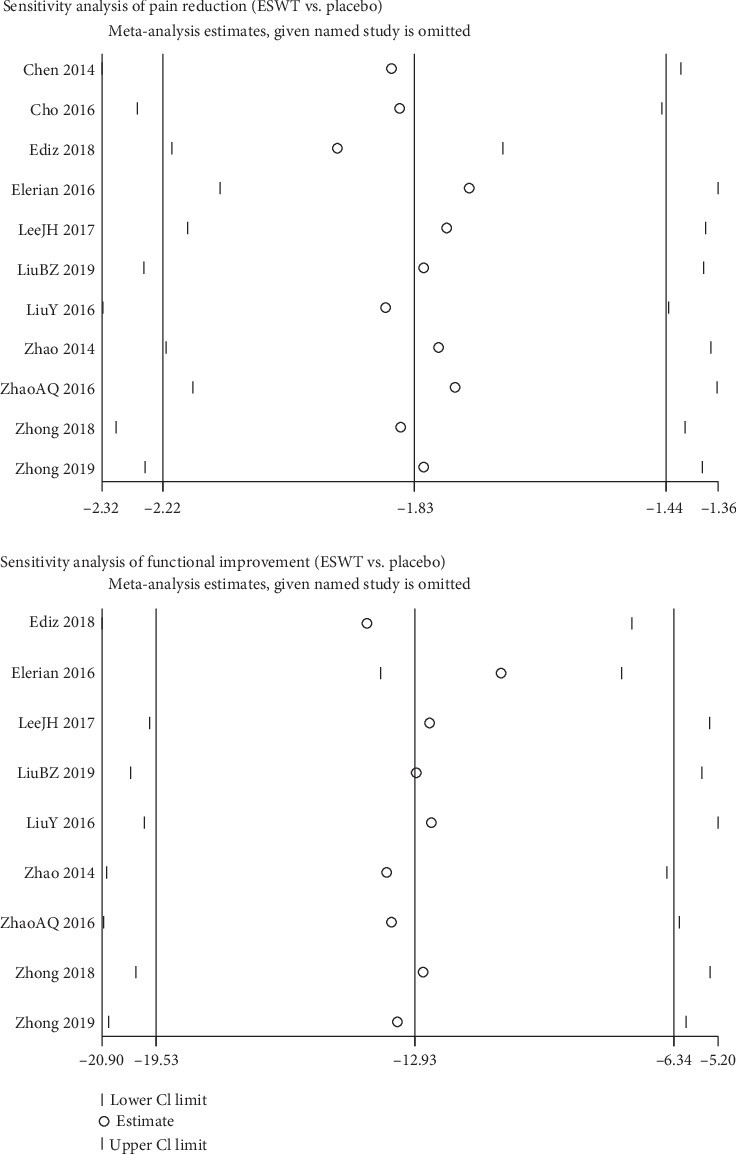
Sensitivity analysis of included studies comparing the ESWT group with the placebo group.

In meta-analysis comparing ESWT with placebo, a single study was excluded each time to evaluate the impact of the individual data on the whole result. The results showed that the pooled effect was robust and no significant deviation from the overall results was detected in our study (Figure 9).

### 3.11. Quality Assessment and Publication Bias

**Figure 10:**
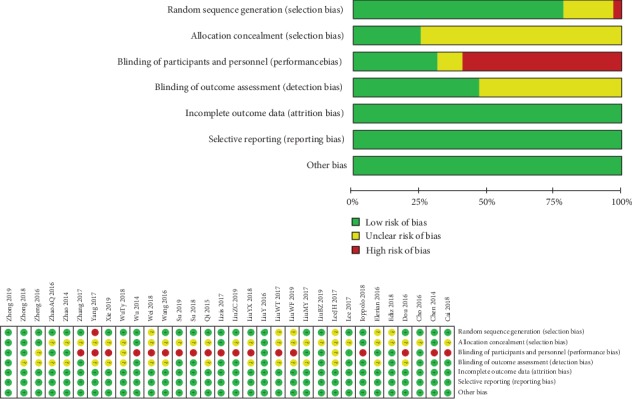
Quality assessment of included articles.

In quality assessment (Figure 10), 19 studies were considered to be high risk in blinding of participants and personnel because the therapeutic properties make it hard to apply blinding. 155 of 224 domains (69.2%) were determined at low risk, and 50 of 224 domains (22.3%) were determined at unclear risk. There was no publication bias in this meta-analysis (pain reduction—Begg's test: *P* = 0.161, Egger's test: *P* = 0.346; functional improvement—Begg's test: *P* = 0.466, Egger's test: *P* = 0.155).

## 4. Discussion

This meta-analysis included 32 studies involving 2408 patients to explore the efficacy and safety of ESWT for the treatment of OA. In this study, the ESWT group showed a statistically significant difference compared with the placebo, corticosteroid, HA, medication, and ultrasound group in both pain reduction and functional improvement, presenting that ESWT might be a successful alternative treatment when above treatments are unavailable. In functional improvement, ESWT showed statistical improvement compared with kinesiotherapy and moxibustion but no statistical difference compared with acupotomy surgery. A significant difference between ESWT and PRP was observed in pain reduction but not in functional improvement. Meanwhile, a statistical difference was found between ESWT and fumigation in functional improvement but not in pain reduction. Additionally, there was no statistically significant difference between ESWT and manipulation in both pain reduction and functional improvement. No serious adverse reaction occurred in all of studies.

Osteoarthritis (OA) is the most common cause leading to musculoskeletal pain [53]. It is considered that the pathological features of OA include articular cartilage destruction, subchondral bone change, osteophyte formation remolding, ligamentous laxity, periarticular muscle weakness, and synovial inflammation, which could result in chronic pain, physical limitation, and joint stiffness [54, 55].

Traditional treatments of OA included nonsurgical therapies and surgical therapies. In the 2014 Osteoarthritis Research Society International guidelines for the management of knee OA, nonsurgical therapies included intra-articular corticosteroids, biomechanical interventions, exercise, education and self-management, weight management, and strength training [56]. Traditional surgical options included joint sparing procedures such as arthroscopic surgery or joint replacing procedures [57]. For treatment, nonsurgical therapy might have limited benefit and could be associated with serious adverse events such as bleeding or gastrointestinal ulcers caused by nonsteroidal anti-inflammatory drugs (NSAIDs) and infection caused by intra-articular injection [58]. As for surgery, it might be inappropriate for aged patients with limiting comorbidities. In such conditions, an effective and safe treatment was needed for patients with OA.

ESWT has been increasingly used in clinical practice over the past few years and shows significant efficacy in some clinical studies [16, 59–61]. It is suggested that ESWT can generate radial or focused pressure waves which bring energy and propagate through tissue [62]. This physical force could stimulate biological effects in a treated area, and the biochemical mechanism of ESWT in OA might be associated with neovascularization, osteogenesis, and chondrogenesis [63–65]. In recent studies, ESWT might lead to upregulation of angiogenic growth factors including endothelial nitric oxide synthase (eNOS) and vessel endothelial growth factor (VEGF), which benefit to neovascularization [66]. ESWT was also found connected with osteogenic transcription factors including VEGF-A and hypoxia inducible factor-1*α* (HIF-1*α*), affecting growth of osteoblasts [67]. Meanwhile, ESWT might elevate levels of nitric oxide (NO), bone morphogenetic protein-2 (BMP-2), protein kinase B (PKB), and transforming growth factor-beta 1 (TGF-*β*1), which facilitate differentiation and proliferation of osteoblasts [68–71]. Also, it was suggested that ESWT could enhance the expression of Pdia-3, a key point of 1*α*,25-dihydroxyvitamin D3 (1*α*,25(OH)_2_D_3_) signaling pathway [72]. This signaling pathway is essential in gene transcription and calcium homeostasis, which was considered beneficial for osteogenesis [73]. Besides, ESWT was revealed to have a dose-dependent effect on the metabolism of mesenchymal stem cells (MSCs), which potentially improve bone regeneration and chondrogenesis [74]. However, the exact mechanism of ESWT is still unknown, and further studies are required for better clinical utilization.

This study also had some limitations. First, we only searched studies in English and Chinese; thus, some potential relative studies in other languages might have been missed. Second, unreported negative results and gray literature could result in publication bias. Third, very few studies compared ESWT with surgery, PRP, and corticosteroid intra-articular injections, traditional Chinese medicine, or kinesiotherapy; thus, the subgroup analysis and sensibility analysis could not be performed, and the outcome might be misleading. Besides, in this meta-analysis, focused ESWT was performed in 8 studies in the experiment group and radial ESWT was administered in 19 studies, while the type of ESWT was unmentioned in the other 5 studies. As a result, it was difficult to perform subgroup analysis according to the different type of ESWT and analyze whether there was a difference between the focused ESWT and radial ESWT in the treatment of OA. Further studies could be carried out to improve this issue.

## 5. Conclusion

In conclusion, ESWT showed a significant effect in the treatment of OA in pain reduction or/and functional improvement compared with placebo, corticosteroid, HA, medication, ultrasound, moxibustion, fumigation, PRP, and kinesiotherapy. However, ESWT failed to show a statistically significant difference compared with manipulation and surgery. As a result, ESWT could be recommended in the treatment of OA as a noninvasive therapy with safety and effectiveness but the grade of recommendations needs to be discussed in a further study.

## Figures and Tables

**Table 1:** Basic characteristics of included studies.

Author	Publication year	Country	Language	Sample size	Control group	Experimental group	Outcome measures	Follow-up time	Type of OA	Type of ESWT
Ediz	2018	Turkey	English	73	Placebo	ESWT 2 times/week Total of 5 weeks	VAS, WOMAC	6 M	Knee	Focused ESWT
Ioppolo	2018	Rome	English	58	HA (3 injections of 0.5 cm^3^ HA) 1 time/week Total of 3 weeks	ESWT 1 time/week Total of 3 weeks	VAS	3 M	Carpometacarpal joint	Focused ESWT
Lee	2017	Korea	English	61	HA (1 injection of 2 mL of HA) 1 time/week Total of 3 weeks	ESWT 1 time/week Total of 3 weeks	VAS, WOMAC	3 M	Knee	Focused ESWT
Lizis	2017	Poland	English	40	KIN 1 time/week Total of 3 weeks	ESWT 1 time/week Total of 5 weeks	WOMAC	5 W	Knee	Unmentioned
Zhao	2013	China	English	70	Placebo	ESWT 1 time/week Total of 4 weeks	VAS, WOMAC	3 M	Knee	Radial ESWT
Liu Y	2016	China	Chinese	86	Placebo	ESWT 1 time/week Total of 8 weeks	VAS, WOMAC	3 M	Knee	Radial ESWT
Liu MY	2017	China	Chinese	158	Medication (celecoxib) oral 200 mg qd 4 weeks	ESWT 1 time/week Total of 4 weeks	VAS, WOMAC	3 M	Knee	Unmentioned
Zhang	2017	China	Chinese	106	Ultrasound 5 times/week Total of 4 weeks	ESWT 1 time/5 days Total of 5 times	VAS	1 M	Knee	Radial ESWT
Zheng	2016	China	Chinese	48	Medication (celecoxib) oral 200 mg qd 4 weeks	ESWT 1 time/week Total of 4 weeks	VAS, WOMAC	1 M	Knee	Radial ESWT
Liu WT	2017	China	Chinese	58	Acupotomy surgery	ESWT 1 time/5 days Total of 6 times	WOMAC	5 W	Knee	Unmentioned
ZhaoAQ	2016	China	Chinese	60	Placebo	ESWT 1 time/week Total of 8 weeks	VAS, WOMAC	2 M	Knee	Unmentioned
Wu	2014	China	Chinese	53	Medication (toricoxi) oral 60 mg qd 4 weeks	ESWT 1 time/week Total of 4 weeks	VAS	6 W	Knee	Radial ESWT
Chen	2014	China	English	120	Placebo Ultrasound 3 times/week Total of 8 weeks	ESWT 1 time/week Total of 6 weeks	VAS	2 M	Knee	Focused ESWT
Lee JH	2017	Korea	English	20	Placebo	ESWT 3 times/week Total of 4 weeks	VAS WOMAC	1 M	Knee	Focused ESWT
Zhong	2019	China	English	63	Placebo	ESWT 1 time/week Total of 4 weeks	VAS WOMAC	3 M	Knee	Radial ESWT
Wang	2016	China	Chinese	78	Massage manipulation 1 time/2 days 10 weeks	ESWT 1 time/5 days Total of 5 weeks	WOMAC	3 M	Knee	Radial ESWT
Zhong	2018	China	Chinese	63	Placebo	ESWT 1 time/week Total of 4 weeks	VAS WOMAC	5 W	Knee	Radial ESWT
Xie	2019	China	Chinese	60	Fumigation bid 3 weeks	ESWT 1 time/week Total of 4 weeks	VAS	3 M	Knee	Radial ESWT
Su	2018	China	Chinese	160	HA (1 injection of 2 mL of HA) 1 time/week Total of 5 weeks	ESWT 1 time/week Total of 5 weeks	VAS WOMAC	5 W	Knee	Radial ESWT
Su	2019	China	Chinese	120	PRP (1 injection of 4 mL of PRP) 1 time/week Total of 5 weeks	ESWT 1 time/week Total of 5 weeks	VAS WOMAC	5 W	Knee	Radial ESWT
Qi	2015	China	Chinese	60	Acupoint moxibustion qd 4 weeks	ESWT 1 time/week Total of 4 weeks	WOMAC	6 M	Knee	Focused ESWT
Yang	2017	China	Chinese	86	Fumigation qd 16 days	ESWT 1 time/5 days Total of 4 weeks	WOMAC	After treatment	Knee	Radial ESWT
Liu ZC	2019	China	Chinese	77	HA (1 injection of 2.5 mL of HA) 1 time/week Total of 5 weeks	ESWT 1 time/week Total of 5 weeks	VAS WOMAC	5 W	Knee	Radial ESWT
Wu TY	2018	China	Chinese	100	Medication (celecoxib) oral 200 mg qd 4 weeks	ESWT 1 time/week Total of 4 weeks	VAS WOMAC	After treatment	Knee	Radial ESWT
Cai	2018	China	Chinese	90	HA (1 injection of z.5 mL of HA) 1 time/week Total of 5 weeks	ESWT 1 time/week Total of 5 weeks	VAS WOMAC	3 M	Knee	Radial ESWT
Wei	2018	China	Chinese	40	Massage manipulation 3 times/week Total of 4 weeks	ESWT 1 time/week Total of 4 weeks	VAS WOMAC	6 W	Knee	Radial ESWT
Cho	2016	Korea	English	18	Placebo	ESWT 1 time/week Total of 3 weeks	VAS	1 W	Knee	Focused ESWT
Liu YX	2018	China	Chinese	72	HA (1 injection of 20 mg of HA) 1 time/week Total of 2 months	ESWT 2 times/week Total of 2 months	VAS WOMAC	2 M	Knee	Unmentioned
Liu BZ	2019	China	Chinese	63	Placebo	ESWT 1 time/week Total of 4 weeks	VAS WOMAC	3 M	Knee	Radial ESWT
Elerian	2016	Egypt	English	60	Placebo corticosteroid injection 1 time/month Total of 2 months	ESWT 1 time/week Total of 3 weeks	VAS WOMAC	2 M	Knee	Radial ESWT
Liu WF	2019	China	Chinese	66	Medication (celecoxib) oral 200 mg qd 4 weeks	ESWT 1 time/week Total of 4 weeks	VAS	After treatment	Knee	Radial ESWT
Dou	2016	China	Chinese	121	Ultrasound	ESWT	WOMAC	1 M	Knee	Focused ESWT
					1 time/2 days for first two intervals 1 time/3 days for 2nd to 6th intervals 1 time/4 days for 6th to 8th intervals					

Abbreviations: RCT: randomized controlled trial; ESWT: extracorporeal shockwave therapy; HA: hyaluronic acid intra-articular injections; PRP: platelet-rich plasma; KIN: kinesiotherapy; VAS: visual analogue scale; WOMAC: Western Ontario and McMaster Universities Osteoarthritis Index; W: weeks; M: months; qd: once a day; bid: twice a day; OA: osteoarthritis.
